# Respiratory Antiviral Immunity and Immunobiotics: Beneficial Effects on Inflammation-Coagulation Interaction during Influenza Virus Infection

**DOI:** 10.3389/fimmu.2016.00633

**Published:** 2016-12-23

**Authors:** Hortensia Zelaya, Susana Alvarez, Haruki Kitazawa, Julio Villena

**Affiliations:** ^1^Immunobiotics Research Group, Tucuman, Argentina; ^2^Institute of Applied Biochemistry, National University of Tucuman, Tucuman, Argentina; ^3^Laboratory of Immunobiotechnology, Reference Centre for Lactobacilli (CERELA-CONICET), Tucuman, Argentina; ^4^Food and Feed Immunology Group, Laboratory of Animal Products Chemistry, Graduate School of Agricultural Science, Tohoku University, Sendai, Japan; ^5^Livestock Immunology Unit, International Education and Research Center for Food and Agricultural Immunology (CFAI), Graduate School of Agricultural Science, Tohoku University, Sendai, Japan

**Keywords:** immunobiotics, influenza virus, inflammation, coagulation, respiratory immunity

## Abstract

Influenza virus (IFV) is a major respiratory pathogen of global importance, and the cause of a high degree of morbidity and mortality, especially in high-risk populations such as infants, elderly, and immunocompromised hosts. Given its high capacity to change antigenically, acquired immunity is often not effective to limit IFV infection and therefore vaccination must be constantly redesigned to achieve effective protection. Improvement of respiratory and systemic innate immune mechanisms has been proposed to reduce the incidence and severity of IFV disease. In the last decade, several research works have demonstrated that microbes with the capacity to modulate the mucosal immune system (immunobiotics) are a potential alternative to beneficially modulate the outcome of IFV infection. This review provides an update of the current status on the modulation of respiratory immunity by orally and nasally administered immunobiotics, and their beneficial impact on IFV clearance and inflammatory-mediated lung tissue damage. In particular, we describe the research of our group that investigated the influence of immunobiotics on inflammation–coagulation interactions during IFV infection. Studies have clearly demonstrated that hostile inflammation is accompanied by dysfunctional coagulation in respiratory IFV disease, and our investigations have proved that some immunobiotic strains are able to reduce viral disease severity through their capacity to modulate the immune-coagulative responses in the respiratory tract.

## Introduction

Influenza virus (IFV) is a member of the *Orthomyxoviridae* family that contains a negative-sense, single-stranded, segmented RNA genome protected by a capsid of viral ribonucleoproteins. This virus is categorized into subtypes based on the expression of hemagglutinin (HA) and neuraminidase on the surface of the viral envelope.

Influenza is a highly contagious viral infection that has a substantial impact on global health and IFV is a major respiratory pathogen that causes a high degree of morbidity and mortality, especially in high-risk populations such as infants, elderly, and immunocompromised hosts. Given the high capacity of IFV to change antigenically, acquired immunity is often not effective to limit infection and therefore vaccination must be constantly redesigned to achieve protection. Improvement of respiratory and systemic innate immune mechanisms has been proposed to reduce the incidence and severity of IFV disease.

In the last decade, several research works have demonstrated that microbes with the capacity to modulate the mucosal immune system (immunobiotics) are a potential alternative to beneficially modulate the outcome of IFV infection. This review provides an update of the current status on the modulation of respiratory immunity by orally and nasally administered immunobiotics, and their beneficial impact on IFV clearance and inflammatory-mediated lung tissue damage. In particular, we describe the research of our group that investigated the influence of immunobiotics on inflammation–coagulation interactions during IFV infection. Studies have clearly demonstrated that hostile inflammation is accompanied by dysfunctional coagulation in respiratory IFV disease, and our investigations have proved that some immunobiotic strains are able to reduce viral disease severity through their capacity to modulate the immune-coagulative responses in the respiratory tract.

## Respiratory Immune Response and IFV

**Figure 1:**
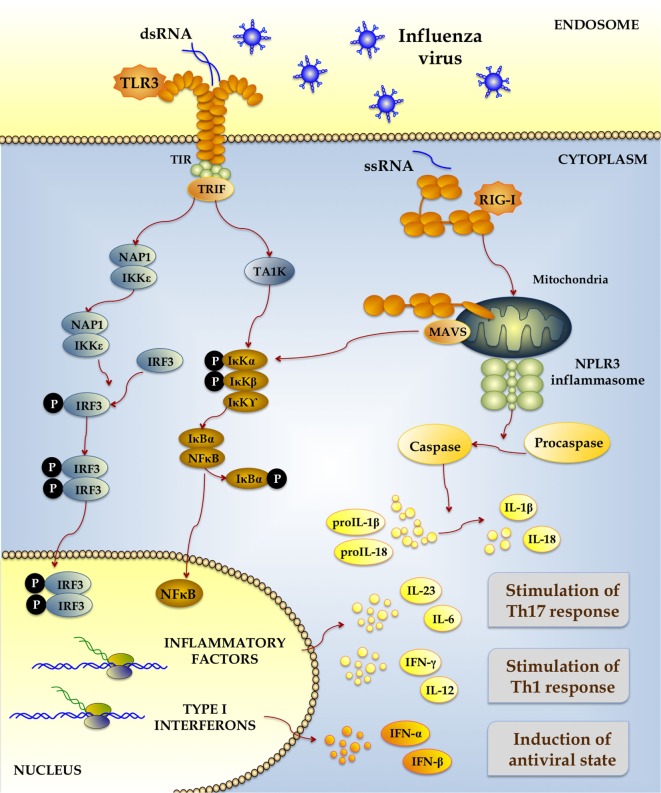
**Signaling pathways activated by the recognition of influenza virus-associated molecular patterns by pattern-recognition receptors expressed in respiratory epithelial cells and immune cells**.

The first barrier that protects the host against IFV infection is the respiratory epithelium through its capacity to recognize the viral attack. When IFV successfully overcomes the respiratory barrier constituted by the mucus layer and the ciliar movement, it mediates its attachment and internalization into respiratory epithelial cells to start its replication (1). During the viral attack, several pathogen-associated molecular patterns (PAMPs) are exposed and recognized by pattern-recognition receptors (PRRs) expressed in respiratory cells (Figure 1). It is now well established that the most important PRRs involved in the recognition of IFV are the Toll-like receptor (TLR)-3 and TLR7 and the RNA recognition protein RIG-1 (2). TLR3 is expressed in endosomes and is able to recognize viral double-stranded RNA (dsRNA) that is produced during viral replication; while endosomal TLR7 and cytoplasmic RIG-I recognize single-stranded RNA (ssRNA). RIG-I signals through mitochondrial antiviral signaling protein. The PAMPs–PPRs interaction leads to the activation of several signaling pathways that induce the activation of nuclear factor κB (NF-κB) and interferon (IFN) regulatory factor 3 (IRF3) and the production of type I and III IFNs and inflammatory cytokines (2).

Type I IFNs, especially IFN-β, produced and released during the earlier stages of IFV infection are key to develop an antiviral state in the respiratory tract. It was reported that human bronchial epithelial cells release preformed IFN-β in response to IFV challenge inducing a protective role (3). IFNs produced by infected cells are able to act in a paracrine or autocrine manner activating their receptors (IFNAR) and increasing the expression of hundreds of genes that counteract viral replication. Functional genomic studies have identified several of the IFN-induced factors that have important roles in controlling IFV replication (2) including the IFN-inducible transmembrane proteins 1, 2, and 3 (4), MX1 proteins (5), and 2′,5′-oligoadenylate synthetase (OAS)-RNAaseL system (6).

Proinflammatory cytokines and chemokines produced as a result of TLR3 and RIG-I activation during IFV infection are also important for the generation of the respiratory antiviral innate immune response. Infection of epithelial cells by IFV increases the expression of TNF-α, IL-6, IL-8, CCL2 (MIP-1), CCL5 (RANTES), CCL3 (MIP-1α), and CXCL10 (IP-10) (7). The production of these cytokines is complemented by activity of inflammasomes that induce the activation of caspase-1 and promote the generation of the active forms of IL-1β and IL-18 (Figure 1). IFV has been shown to activate mainly the NLRP3 inflammasome which is essential for the protection against the virus since several studies demonstrated that mice lacking NLRP3 or caspase-1 have decreased IL-1β and IL-18 secretion and increased mortality after IFV challenge (8–10).

**Figure 2:**
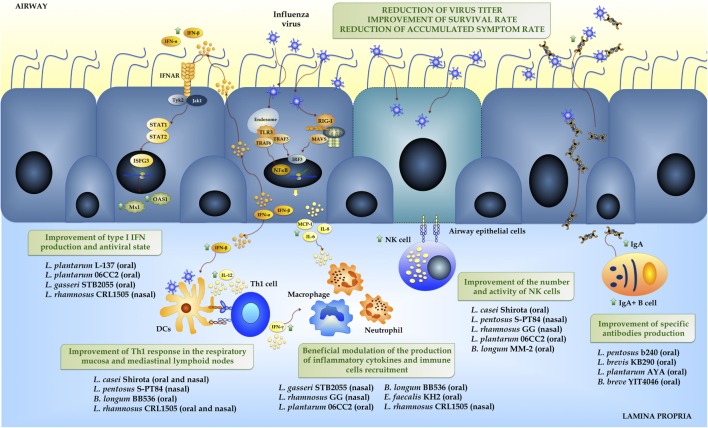
**Innate immune response against influenza virus in the respiratory mucosa mediated by the recognition of viral-associated molecular patterns by pattern-recognition receptors expressed in respiratory epithelial cells**. Beneficial effects of immunobiotics administration on the resistance and immune response against Influenza Virus in the respiratory mucosa.

The proinflammatory cytokines and chemokines are responsible for the activation of resident immune cells such as innate lymphoid cells, alveolar macrophages, and dendritic cells (DCs) as well as for the recruitment of neutrophils, macrophages, and lymphocytes into the respiratory tract (2, 7) (Figure 2). Respiratory cells infected with IFV express HA on their surface that is important for its recognition by NK cells (11). It was established that HA expressed by the infected cells is recognized by NKp44 and PKp46 receptors of NK cells that then mediated the lysis of IFV-infected cells (12). Macrophages activated during IFV infection produce IFNs, IL-6, TNF-α, and nitric oxide synthase that amplify the inflammatory response. In addition, macrophages limit the viral spread by the elimination of apoptotic-infected cells and through phagocyte-mediated opsonophagocytosis of IFV (7). The production of proinflammatory cytokines during the generation of the respiratory innate immune response against IFV also conditions the adaptive immune response, which includes the production of virus-specific systemic and mucosal antibodies as well as the induction of specific T cell responses (13).

After exposure to IFV there is an activation of antibody responses in the respiratory tract. Upper airway exposure results primarily in an IgA response while the contact of IFV with the deep lung induces an increased production of pathogen-specific IgG (14). Following exposure to IFV in the airways there is an antigen uptake and processing by DCs, activation of CD4^+^ Th cells, and generation of IgA-producing plasma cells that populate airway lamina propria. Secretory IgA has a non-inflammatory protective function since these antibodies can bind to virus without activating complement or stimulating the release of inflammatory mediators by innate immune cells (14, 15). IgA prevents IFV from adhering to the epithelial surface by inducing viral agglutination, and masking adhesion epitopes. In the deep lung, when IFV reach the alveolar space, there is a differentiation and expansion of antibody-secreting plasma cells that are committed to the production of IgG. Induction of neutralizing respiratory and serum IgG antibodies is a key event in the defense against influenza infection since IgG prevents systemic spread (16). Influenza infection in the lungs also activates the cellular adaptive immune response by stimulating the production of IFN-γ by Th1 cells that effectively activate CD8^+^ T cells and macrophages, which clear virus and infected cells from the lungs (17). Therefore, during uncomplicated influenza, adaptive immune response ultimately results in clearance of IFV from the lungs through the activity of virus-specific antibodies and CD4^+^ and CD8^+^ T cells.

## Role of Microbiota on IFV Infection

The gut microbiome, which is defined as the collective group of microorganisms and their associated genes within the intestinal tract, is considered as a key player in the modulation of host intestinal immune responses (18, 19). In fact, the impact of gut commensal bacteria on the innate and adaptive immune responses to enteric pathogens has been recognized conclusively (20–22). However, the effect of gut microbiome on the immune responses in distal mucosal sites and its impact in the outcome of respiratory infections has recently been exposed. In this regard, some studies have demonstrated an important role for intestinal microbiota in maintaining respiratory antiviral immunity against IFV (23, 24).

Iwasaki and colleagues observed that commensal bacteria within the gut, especially gram-positive bacterial populations, had an important role in supporting an appropriate immune response to IFV infection in the respiratory tract (23). The work demonstrated that oral antibiotic treatments impaired the resistance of mice to the intranasal infection with IFV as noted by the elevated lung viral titers when compared to non-antibiotic-treated animals. Results indicated that gut gram-positive bacteria provided protection by triggering an adequate inflammatory response through inflammasomes activation. In antibiotic-treated mice, synthesis of pro-IL-1β, pro-IL-18, and NLRP3 was impaired even at the steady state. In addition, depletion of gram-positive bacterial populations in the gut resulted in an alteration of the distribution and activation of respiratory DCs at steady state as well as in a diminished DCs migration from the lung to the draining lymph nodes, resulting in reduced activation of CD8^+^ and CD4^+^ T cells after influenza challenge (23). Alteration of respiratory DCs activities also correlated with impaired expansion of influenza-specific B cells and reduced influenza-specific antibodies.

By using germ-free and antibiotic-treated mice challenged with IFV, Abt et al. (24) showed that the absence or the alteration of intestinal microbiota induced an exacerbated weight loss, a greater drop in blood oxygen saturation, increased mortality, and elevated lung viral titers indicating a weaker ability to resist influenza. Even more, germ-free and antibiotic-treated mice infected with IFV experienced higher epithelial cell necrosis, peribronchiolar inflammation, severe bronchiole epithelial degeneration, and epithelial hyperplasia when compared to conventional animals (24). Interestingly, those effects were observed when both the PR8 strain and the X31-GP33 virus, a less pathogenic strain of IFV that causes minimal mortality and morbidity in conventional mice, were used. Consistent with the work by Ichinohe et al. (23), germ-free and antibiotic-treated mice challenged with IFV had an impaired adaptive immune response as shown by the lower influenza-specific antibodies (serum IgM and IgG), fewer number of IFV-specific T cells present in lungs, as well as a reduced capacity of specific T cells to produce effector cytokines such as TNF-α, MIP-1α, IL-2, and IFN-γ (24). Moreover, authors demonstrated that the alterations of adaptive immune responses were related to defects in the early innate immune response mediated by macrophages. In fact, transcriptional profiling and computational analyses of macrophages from antibiotic-treated mice indicated a reduced expression of antiviral genes including *Ifnb, Tnfa, Il1b, Irf7, Mx1*, and *Oas1a* when compared to conventional mice. In addition, functional assays of macrophages from antibiotic-treated mice demonstrated that those cells had a defective response to type I IFNs and an impaired capacity to limit IFV replication (24).

The cellular and molecular mechanisms through which the gut microbiome and their derived signals maintain and modulate immune responses in distal mucosal sites are poorly understood. Two possible mechanisms that are not mutually exclusive have been proposed to explain this beneficial effect of the gut microbiome. One possibility is that distal mucosal and peripheral immune cells are directly exposed to bacterial products that activate PRRs in the steady state and help to maintain the normal immune tone. There is evidence that bacterial products from gut commensals such as peptidoglycan can be absorbed and circulate throughout the host and help to modulate the normal development of immune cells (25). In line with this hypothesis, Iwasaki and colleagues speculated that bacterial products from gut commensals trigger PRRs to stimulate immune cells systemically and that factors released by those cells supported steady-state production of pro-IL-1β, pro-IL-18, and NLR proteins. This idea was sustained by their observation that intestinal injection of TLR ligands restored immune responses to IFV in antibiotic-treated mice (23). Another possibility is that commensal bacteria may indirectly influence systemic and distal mucosal immune responses through immune factors released from the intestinal mucosa including cytokines, chemokines, and grow factors.

These research works demonstrated that the gut microbiome provides signals to sustain antiviral innate immune defense mechanisms in the respiratory tract allowing robust and efficient effector responses upon challenge by viral pathogens such as IFV. Therefore, the role of the gut microbiome in regulating respiratory antiviral immunity represents an exciting area of research that could help to provide the scientific basis for the development of novel prevention strategies for lung infectious diseases. However, several questions need to be answered to identify new alternatives to improve antiviral respiratory defenses by modulating the microbiota. How the different microbial species from the gut microbiota influence the common mucosal immune system? Which PRRs are activated by the gut microbiota to functionally modulate antiviral immunity locally and in distal mucosal sites? Which cellular functions are modulated by the microbiota after PRR activation? Has the microbiota the ability to influence immune responses to other respiratory viruses? Are similar immune mechanisms activated by the microbiota in high-risk populations (infants, elderly, immunocompromised hosts) in which respiratory viral infections are more frequent and severe? Is it possible to beneficially modulate antiviral respiratory defenses by orally administering selected microorganisms with immunomodulatory capacities? Research from the last years has provided some answers for the last question.

## Beneficial Effects of Immunobiotics on IFV Infection

The first studies that assessed the capacity of immunobiotics to favorably modulate the immune response against IFV focused on the humoral immunity (Table 1). Yasui et al. (26) reported that the oral administration of *Bifidobacterium breve* YIT4064 improved the production of anti-IFV IgG antibodies in serum of IFV-infected mice. The YIT4064 strain reduced viral titers, improved the survival rate, and decreased the severity of the symptoms associated to the influenza infection. Similarly, it was shown that orally administered non-viable *Lactobacillus pentosus* b240 (27) or viable *Lactobacillus brevis* KB290 (28) were able to improve the levels of respiratory specific IgA and IgG antibodies of mice challenged with IFV. Moreover, the improved humoral response induced by these strains correlated with significant reduction of viral titers, body weight loss, and a decrease of the alterations of physical conditions induced by IFV. More recently, Kikuchi et al. (29) demonstrated a beneficial effect on the outcome to IFV infection related to an improved respiratory humoral response in *Lactobacillus plantarum* AYA-treated mice. In addition, the work proposed a mechanism for the distal immunomodulatory activity induced by orally administered immunobiotics. Authors showed that *L. plantarum* AYA fed to mice impacted in Peyer’s patches (PPs) inducing an activation of antigen presenting cells (mainly CD11b^+^ DCs) and increasing the production of IL-6. Those changes promoted an IgM-to-IgA class switch recombination, the differentiation of IgA^+^ B cells into plasma cells, and improved the production of mucosal IgA in both the intestine and the respiratory tract. Those studies show that immunobiotics are capable to modulate the production of systemic and mucosal antibodies against influenza and therefore, to enhance the humoral immune response (Figure 2). However, the precise mechanism by which orally administered immunobiotics induce IgA production in distant mucosal sites remains unclear.

**Table 1:** **Effect of immunobiotics on influenza virus (IFV) infection in mice models**.

Immunobiotic strain	Viability	Administration protocol	Challenge	Mice	Immunobiotic effects	Effect on IFV infection	Reference
**Effects on humoral immune response**							
*Bifidobacterium breve* YIT4064	Non-viable	Oral *ad libitum* administration of food with 0.05% *B. breve* YIT4064 during 15 weeks before IFV challenge. Treatment was continued for 2 weeks after infection	IFV (H1N1) strain A/PR/8/34	Six-week-old male BALB/c	Improved the production of anti-IFV IgG antibodies in serum	Reduced viral titers, improved survival rate, and decreased severity of symptoms	(26)
*Lactobacillus pentosus* b240	Non-viable	*L. pentosus* was administered by gavage at doses of 0.4, 2, or 10 mg per mouse per day during 21 days before IFV challenge. Treatment was continued for 2 weeks after infection	IFV (H1N1) strain A/PR/8/34	Six-week-old female BALB/c	Improved levels of respiratory IgA and IgG specific antibodies	Reduced IFV titers	(27)
*Lactobacillus brevis* KB290	Viable	*L. brevis* was administered by gavage at a dose of 10^9^ cells per mouse per day during 14 days before IFV challenge	IFV (H1N1) strain A/PR/8/34	Six to eight-week-old female BALB/c	Improved levels of IFV-specific IgA in the respiratory tract	Reduced body weight loss and decreased alterations of physical conditions	(28)
*Lactobacillus plantarum* AYA	Non-viable	Oral *ad libitum* administration of food with 5% of *L. plantarum* AYA (120 mg per mouse per day) during 28 days before IFV challenge	IFV (H3N2) strain X-31	Seven-week-old female BALB/c	Improved the production of IgA in the respiratory tract	Reduced body weight loss and decreased mortality	(29)
**Effects on cellular immune response**							
*Lactobacillus casei* Shirota	Non-viable	Oral *ad libitum* administration of food with 0.05% of *L. casei* Shirota during 4 weeks before IFV challenge	IFV (H1N1) strain A/PR/8/34	Fifteen-week-old female BALB/c	Improved systemic and respiratory NK cell activity and production of interferon (IFN)-γ and TNF-α by respiratory lymphocytes	Reduced IFV titers	(30)
*L. casei* Shirota	Viable	*L. casei* Shirota was administered by gavage at a dose of 10^9^ cells per mouse 5 times/week for about 3 weeks (total, 17 times) before IFV challenge	IFV (H1N1) strain A/PR/8/34	Neonatal and infant mice	Improved systemic and respiratory NK cell activity and production of IFN-γ and TNF-α by respiratory lymphocytes	Reduced IFV titers, decreased accumulated symptom rate, and decreased mortality	(31)
*Lactobacillus gasseri* TMC0356	Viable	Ten milligrams of lyophilized bacteria in 200 µl of saline was administered orally per day during 14 days before IFV challenge. Treatment was continued for 5 days after infection	IFV (H1N1) strain A/PR/8/34	Five-week-old female BALB/c	Improved NK cell activity and production of IFN-γ	Reduced virus titers and diminished lung pathological changes	(32)
*Lactobacillus rhamnosus* GG	Viable	Ten milligrams of lyophilized bacteria in 200 µl of saline was administered orally per day during 14 days before IFV challenge. Treatment was continued for 5 days after infection	IFV (H1N1) strain A/PR/8/34	Five-week-old female BALB/c	Improved NK cell activity and production of IFN-γ	Reduced virus titers and diminished lung pathological changes	(32)
*L. plantarum* 06CC2	Non-viable	*L. plantarum* was administered by gavage twice daily during 2 days before IFV challenge (20 mg/mouse). Treatment was continued for 7 days after infection	IFV (H1N1) strain A/PR/8/34	Six-week-old female BALB/c	Beneficially modulated NK cells activity and improved Th1 response	Reduced virus titers and diminished lung pathological changes	(33)
*Bifidobacterium longum* MM-2	Viable	Oral administration of 2 × 10^9^ cells per mouse per day during 14 days before IFV challenge. Treatment was continued for 2 days after infection	IFV (H1N1) strain A/PR/8/34	Six-week-old female BALB/c	Increased respiratory NK cell activity and IFN-γ production	Improved clinical symptoms, reduced mortality, and decreased virus titers	(34)
*L. casei* Shirota	Non-viable	Nasal administration of 20 or 200 µg per mouse per day during 3 days before IFV challenge	IFV (H1N1) strain A/PR/8/34	Ten to eleven-week-old female BALB/c	Increased levels of IL-12, IFN-γ, and TNF-α in mediastinal lymphoid nodes and lungs	Decreased virus titers and increased survival rates	(35)
*L. pentosus* S-PT84	Non-viable	Nasal administration of 20 or 200 µg per mouse per day during 3 days before IFV challenge	IFV (H1N1) strain A/PR/8/34	Eight to twelve-week-old female BALB/c	Increased IL-12, IFN-α, and NK cell activity in the respiratory tract. Increased levels of IL-12 and IFN-γ in mediastinal lymphoid nodes	Decreased virus titers and increased survival rates	(36)
*L. rhamnosus* GG	Viable	Nasal administration of 200 µg per mouse per day during 3 days before IFV challenge	IFV (H1N1) strain A/PR/8/34	Seven-week-old female BALB/c	Increased respiratory NK cell activity	Reduced IFV titers, decreased accumulated symptom rate, and increased survival rates	(37)
*L. rhamnosus*	Viable	Sublingual administration of 10^8^ cells per mouse per day during 7 days before IFV challenge	IFV (H1N1) strain A/FMI/33	Adult female BALB/c	Improved levels of IgA specific antibodies, IL-12, and decreased levels of TNF-α and IL-6 in lungs. Increased NK cell activity in spleen. Increased CD25 expression by CD4^+^ and CD8^+^ in lung and mediastinal lymphoid nodes	Increase of the survival rates and decrease in the lung lesion scores	(38)
**Effects on innate immune response**							
*L. plantarum* L-137	Non-viable	Intragastric administration of 5–100 mg/kg of mouse per day during 7 days before IFV challenge. Treatment was continued for 7 days after infection	IFV (H1N1) strain A/NWS/47	Seven-week-old female C57BL/6	Improved production of type I IFNs	Reduced viral loads in lungs and improved survival	(39)
*L. gasseri* SBT2055	Viable	Oral administration of 10^8^ or 10^9^ cells per mouse per day during 7–21 days before IFV challenge	IFV (H1N1) strain A/PR/8/34	Five to seven-week-old male C57BL/6	Enhanced lung expression of the antiviral genes *Mx1* and *Oas1a* and differentially regulated inflammatory response	Enhanced survival rates, reduced lung viral titers and diminished lung inflammatory damage	(40)
*L. rhamnosus* CRL1505	Viable	Oral administration of 10^8^ cells per mouse per day during 5 days before IFV challenge	IFV (H1N1) strain A/PR/8/34	Six-week-old male BALB/c	Differentially regulated levels and kinetics of inflammatory cells (neutrophils and macrophages) and cytokines (TNF-α, IL-6, IL-10, and type I IFNs) Diminished coagulation activation in blood and respiratory tract	Decreased IFV titers in lungs, lessened pulmonary damage, and increased survival	(41)
*L. rhamnosus* CRL1505	Viable and non-viable	Nasal administration of 10^8^ cells per mouse per day during 2 days before IFV challenge	IFV (H1N1) strain A/PR/8/34	Six-week-old male BALB/c	Differentially regulated levels and kinetics of inflammatory cells (neutrophils and macrophages) and cytokines (TNF-α, IL-6, IL-10, and type I IFNs) Diminished coagulation activation in blood and respiratory tract	Decreased IFV titers in lungs, lessened pulmonary damage, and increased survival	(42)
*Enterococcus faecalis* KH2	Viable and non-viable	Intragastric administration of 8.5 × 10^10^ cell per kg of mouse per day during 7 or 12 days before IFV challenge	IFV (H1N1) strain A/WSN/33	Adult male C57BL/6	Diminished concentrations of proinflammatory factors especially MCP-1	Reduced mortality, weight loss, and lung viral titers	(43)
*L. pentosus* b240	Non-viable	Oral administration of 10^10^ cells per mouse per day during 21 days before IFV challenge. Oral treatment was continued for 14 days after infection	IFV (H1N1) pdm strain A/California/04/09	Six-week-old female BALB/c	Downregulated expression of the immune related genes *Cyr61, Egr1*, and *Fos*, and genes related to Acyl-CoA-mediated metabolism. Upregulated expression of the antiviral gene Rsad2 in the lungs	Prolonged mouse survival. No effect on virus titers and no apparent differences in the extent of lung damage	(44)

It was also demonstrated that immunobiotics are able to improve cellular immune response against IFV (Figure 2). In this regard, it was reported that orally administered *Lactobacillus casei* Shirota improved the outcomes of IFV infection of aged (30) and infant mice (31) by increasing systemic and respiratory NK cell activity and improving the production of IFN-γ and TNF-α by respiratory lymphocytes. Both studies also demonstrated that IFV titers were significantly reduced in aged and infant mice treated with the Shirota strain (30, 31). Similar to the mechanism proposed to explain the improvement of humoral response, it was postulated that immunobiotic *L. casei* Shirota stimulated Th1 cells and NK cells in PPs and induced a mobilization of those cells to lungs and respiratory-associated lymphoid tissues where they produced IFN-γ and enhanced the antiviral defenses. Several other studies corroborated these findings by showing similar effects for orally administered lactobacilli (32, 33). Immunobiotic *Lactobacillus* strains (*L. gasseri* TMC0356, *L. rhamnosus* GG, or *L. plantarum* 06CC2) beneficially modulated NK cells activity and Th1 response against IFV, diminished virus titers and reduced lung pathological changes (32, 33) (Table 1). More recently, Kawahara et al. (34) described the improvement of respiratory antiviral response by an orally administered bifidobacteria strain. It was shown that *Bifidobacterium longum* MM-2 increased respiratory NK cell activity and IFN-γ production resulting in improved clinical symptoms, reduced mortality, and decreased virus titers after IFV challenge.

Research work has also demonstrated that nasal administration of immunobiotics is an interesting alternative to improve cellular response against influenza infection (35–37) (Table 1). Hori et al. (35) observed that BALB/c mice nasally treated with non-viable *L. casei* Shirota had increased levels of IL-12, IFN-γ, and TNF-α in mediastinal lymphoid nodes and lungs. This improved cellular respiratory immunity correlated with a beneficial clinical outcome to IFV challenge. Similar observations were performed with nasally administered *L. pentosus* S-PT84 (36) and *L. rhamnosus* GG (37).

Other recent studies have also demonstrated the ability of immunobiotics to improve respiratory innate antiviral defenses in the respiratory tract (Table 1; Figure 2). It was reported that orally administered non-viable *L. plantarum* L-137 improved protection against IFV by increasing type I IFN production (39). The work clearly demonstrated that the increased production of IFN-β induced by the immunobiotic strain correlated with the reduction of viral loads in lungs as well as the improved survival of infected mice. More recently, it was shown that *L. gasseri* SBT2055 enhanced survival rates and reduced lung viral titers in mice infected with IFV (40). Interestingly, authors observed that the lung expression of the antiviral genes *Mx1* and *Oas1a* was enhanced in *L. gasseri* SBT2055-treated mice and that the inflammatory response triggered by IFV was differentially regulated inducing a lower inflammatory damage (40).

Our group has also reported a beneficial regulation of the IFV-triggered inflammatory response by immunobiotics. Lung damage induced by IFV is known to be produced by virus replication as well as the uncontrolled inflammatory response that is characterized by a hypersecretion of proinflammatory cytokines, especially TNF-α, IL-1β, and IL-6 (45). The adequate production of inflammatory factors is necessary to protect against IFV infection together with an appropriate regulation with anti-inflammatory cytokines to prevent the damage of lung tissue. Thus, the proper balance of cytokines is a key factor in determining the outcome of IFV infection. In this regard, we observed that orally (41) or nasally (42) administered immunobiotic *L. rhamnosus* CRL1505 differentially regulated the levels and kinetics of inflammatory cells and cytokines in mice after IFV challenge. In our experimental model, we observed increased levels of respiratory TNF-α, IL-6, neutrophils, and macrophages in CRL1505-treated mice early after the challenge with IFV. Later, proinflammatory cytokines and infiltrated cells started to decrease in immunobiotic-treated animals in contrast to control mice, in which those parameters continued increasing. The trend toward lower inflammatory factors and cells registered later during IFV infection in *L. rhamnosus* CRL1505-treated mice correlated with a reduced severity of pulmonary damage when compared to control mice (41, 42).

Chen et al. (43) also investigated the ability of orally administered *Enterococcus faecalis* KH2 to beneficially modulate the innate immune response to influenza infection. Authors observed that KH2 strain protected C57BL/6 mice against IFV as observed by the reduced mortality, weight loss, and lung viral titers. As expected, IFV enhanced the levels of proinflammatory mediators in the respiratory tract including IL-6, TNF-α, IFN-γ, IL-1β, IL-17, and MCP-1 while the treatment with *E. faecalis* significantly diminished the concentrations of proinflammatory factors, especially MCP-1. Considering that monocyte migration mediated by MCP-1 has been linked to several respiratory inflammatory disorders including IFV infection, authors investigated the role of MCP-1/CCR2 pathway in the immunobiotic effect of *E. faecalis* KH2. The work reported that the protective activity of the KH2 strain was abrogated when recombinant MCP-1 was administered concomitantly (43).

It is not clear how immunobiotics initiates the cross-talk with the immune system in order to modulate the respiratory antiviral immunity. It is not known exactly which PRRs are activated by immunobiotics in the intestinal or respiratory mucosa to functionally modulate antiviral immunity locally and in distal mucosal sites, respectively. Neither it has been determined with exactitude which cellular functions are modulated by immunobiotics immediately after PRR activation. Research from the last decade has demonstrated that the immunomodulatory effects of probiotic bacteria are the consequence of complex interactions between several bacterial molecules and host receptors located in different immune and non-immune cells (46, 47). It has also been shown that the immunomodulatory properties of immunobiotics are dependent on the strains. Therefore, studies carried out with certain strains cannot be easily extrapolated to other bacteria, even those of the same genus and species (48, 49). Consequently, it is still necessary to carry out deeper studies to find out the molecular mechanisms by which immunobiotics beneficially influence the respiratory antiviral immunity.

The studies mentioned before showed the potential of immunobiotics to be used for the reduction of the incidence and severity of IFV infections. However, in addition to deepening the knowledge of their mechanisms of action, several other points should be considered for the efficient application of immunobiotics in humans.

For example, it is necessary to determine the best time as well as the most appropriate route for their administration. Immunobiotics used as components of functional foods can be included in diets on a regular basis and thus help to improve respiratory defenses, especially in high-risk populations and during the seasons with the highest incidence of respiratory infections occurs. In this sense, in a randomized controlled trial we demonstrated that *L. rhamnosus* CRL1505 (administered in a yogurt formulation) improved mucosal immunity and reduced the incidence and severity of intestinal and respiratory infection in children (50). Hence, the incidence of infectious events was reduced from 66% in the placebo group to 34% in the group that received the probiotic yogurt. Furthermore, there was also a significant reduction in the occurrence of indicators of disease severity such as fever and the need for antibiotic treatment in children receiving the probiotic yogurt. This immunobiotic yogurt (YOGURITO^®^) has been included into official National Nutritional Programs in Argentina and is given daily to children at schools in several provinces thanks to the Government actions. Epidemiological studies in the schools receiving the immunobiotic product have shown a reduction in the incidence of infections and in the associated school absenteeism (Alvarez et al., unpublished results).

On the other hand, as mentioned earlier the nasal administration of immunobiotics is more efficient than the oral administration to enhance respiratory immunity. This route of administration poses a practical disadvantage considering that the treatments with immunobiotics showed favorable results when they were used before the infectious challenges. In this way, it would be necessary to predict the exact moment in which the viral pathogen will be in contact with the host in order to carry out the prophylactic immunobiotic treatment. This option could be used for example during a school or work outbreak in which cases of respiratory infections occur and it is desired to prevent or reduce the severity of infections in asymptomatic individuals. For an intervention of these characteristics, it would be also important to determine the exact time after the contact with the virus in which it is possible to administer immunobiotics to achieve the beneficial effect. In a recent study, Percopo et al. (51) have defined this as “the window of opportunity.” The work evaluated the effect of the nasal administration of live or inactivated *L. plantarum* NCIMB 8826 in a mice model of severe respiratory infection with the pneumonia virus of mice (PVM) and found that immunobiotic treatment promoted full survival from acute PVM infection when administered within 1 day after virus challenge (51). Similar studies would be of value in IFV infection models.

Another point of interest is related to the duration of the improvement of respiratory defenses after the last immunobiotic administration. Our studies have showed that the immunomodulatory effect of some nasally administered immunobiotics persisted for at least 15 days (Villena et al., unpublished results). Other studies have also reported short-term protection after nasal treatment with different immunobiotic strains (43). Interestingly, Garcia-Crespo et al. (52) found that adult mice primed nasally with *L. plantarum* NCIMB 8826 or *Lactobacillus reuteri* F275 were completely protected against lethal PVM infection and that protection persisted for at least 5 months after the initial priming. These findings open an interesting challenge in the study of immunobiotics to improve the defenses against IFV, since it would be very useful to establish the duration of the protective effect for each strain and treatment, since in the majority of cases these long-term studies were not taken into account.

IFV infections often result in mild to moderate lung infection; however, life-threatening disease can occur. It has been demonstrated that the most severe disease outcomes are associated with secondary bacterial pneumonia caused primarily by *Staphylococcus aureus* or *Streptococcus pneumoniae* (53). Taking into account the high incidence of viral infections and the frequency of associated secondary bacterial infections which contribute to aggravate the health status of the host and reduce its chance of recovery, various approaches for preventing and treating influenza and secondary bacterial pneumonia are been investigated. A wide range of antibiotics and anti-inflammatory drugs has been tested in mice [reviewed in Ref. (54)]. It would be of interest to evaluate the potential beneficial effect of immunobiotics on these circumstances. In this regard, preliminary studies from our laboratory showed that nasally administered *L. rhamnosus* CRL1505 is able to improve survival, reduce bacterial cell counts in lung and blood, and limit lung inflammatory damage caused by *S. pneumoniae* infection in mice produced after the infection with IFV or respiratory syncytial virus (RSV) (Villena et al., unpublished results). These results opened an interesting topic for future investigations.

Finally, it would be also of interest to investigate whether immunobiotic treatments may influence other physiological systems involved in the defenses against viral respiratory infections such as the coagulation system. Our group has made some progress in this regard, as mentioned below.

## Respiratory Immune-Coagulative Response and IFV

Coagulation is an extremely ordered process that involves the interaction of three key components: endothelial cells (ECs), platelets, and coagulation factors. Tissue injury that activates ECs typically initiates coagulation that is characterized by the binding of platelets to activated ECs and the formation of the platelet plug. Almost simultaneously, tissue factor (TF) released by ECs result in factor X activation, which induces thrombin and the generation of fibrin strands to strengthen the platelet plug leading to a stable platelet–fibrin clot. All these processes are tightly regulated by anticoagulant and fibrinolytic mechanisms to avoid thrombotic and/or haemorrhagic complications. A key role has been attributed to ECs in the temporal and special regulation of coagulation activation. Resting ECs avoid the inappropriate plug formation by controlling platelet adhesion and activation and generating several anticoagulant factors providing a non-thrombogenic barrier (55, 56). Once activated or injured, ECs expose collagen to blood, increase platelet binding and aggregation, reduce the expression physiological anticoagulant factors, increase the expression of TF and von willebrand factor, and suppress the fibrinolytic activity (57, 58). All these changes in the hemostatic system facilitate thrombosis in the infected or inflammated tissue.

**Figure 3:**
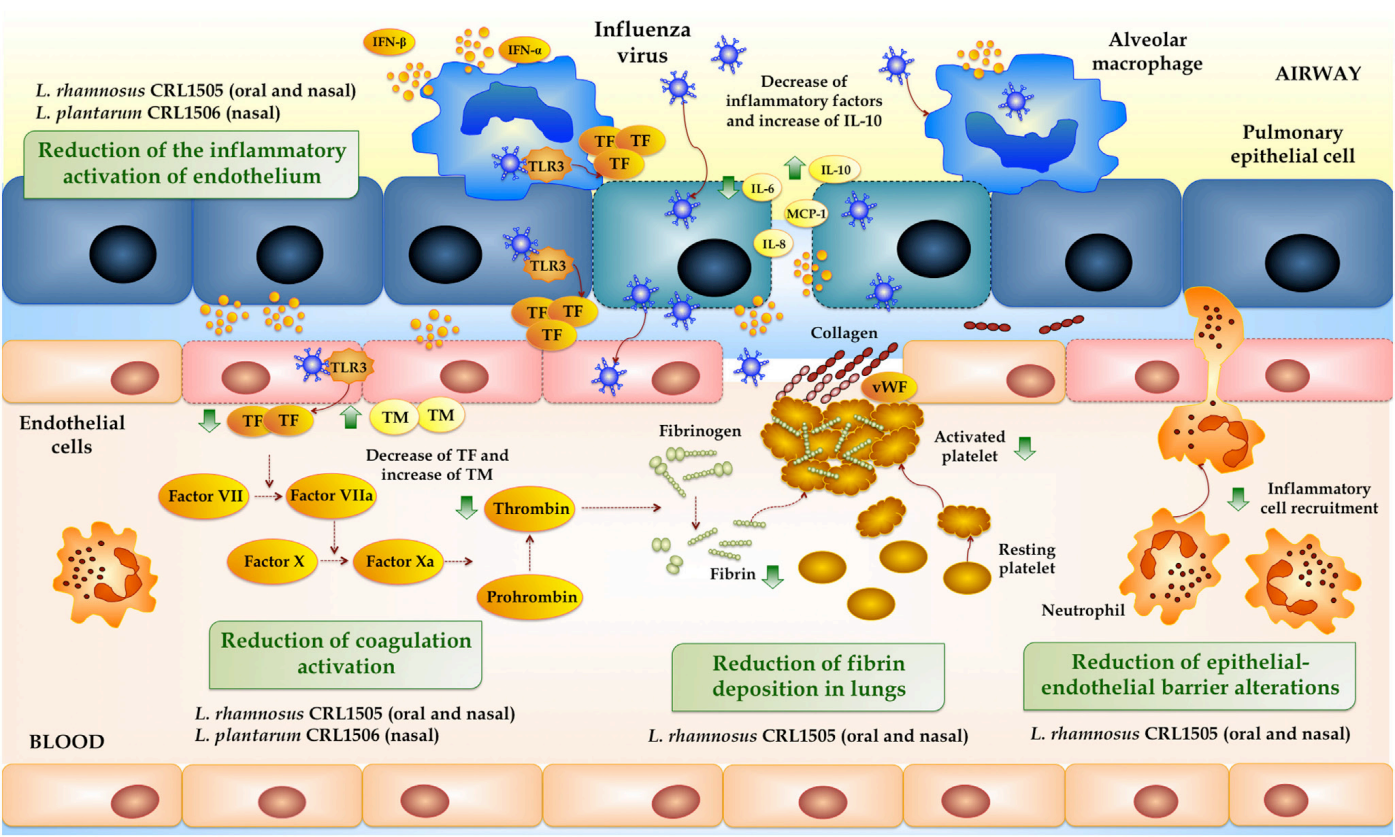
**Activation of the immuno-coagulative response by influenza virus in the respiratory mucosa**. Beneficial effects of immunobiotics administration on the immuno-coagulative response triggered by toll-like receptor 3 or Influenza Virus in the respiratory mucosa.

Both hemorrhagic and thrombotic complications have been described during IFV infection. Influenza is able to cause pulmonary hemorrhage and edema related to coagulopathy or induce uncontrolled thrombosis through an over-activated coagulation (Figure 3) (55, 58). Animal models have helped to explain the mechanisms by which IFV infection activates coagulation and key role has been attributed to TF. It was described that IFV activates coagulation by enhancing TF production, thrombin generation and fibrin deposition in C57BL/6 mice (59). In a mice model of IFV infection, it was recently shown that wild-type animals increased lung TF expression and activation of coagulation but presented alveolar hemorrhage (60). Moreover, selective deletion of TF in epithelial cells from lung significantly reduced TF expression after IFV infection and had higher alveolar hemorrhage and reduced survival than controls. On the contrary, deficiency of TF in either respiratory myeloid cells or ECs did not enhanced alveolar hemorrhage or modified survival of IFV-infected mice (60). These results indicate that an appropriate modulation in the production of TF in the lung during IFV infection is necessary to maintain tissue hemostasis avoiding hemorrhage and excessive fibrin deposition. Production of TF by lung epithelial cells will be required to maintain alveolar hemostasis during IFV infection, while excessive release of TF by macrophages and ECs would contribute to pathology and lung tissue injury (59, 60).

It is considered that ECs may play an important role in the pathogenesis of IFV. Influenza infection is able to induce alveolar edema and pulmonary hemorrhage through the alteration of ECs via several mechanisms, including direct damage and loss of tight junctions and apoptosis (61). In addition, recognition of damage-associated molecular patterns such as HMGB1 or oxidized phospholipids through TLR4 activates ECs to drive lung injury (62). Direct stimulation of TLR3 by viral RNA also results in the upregulation of TF and the downregulation of thrombomodulin (TM) in ECs (63). At the same time, the inflammatory activation of ECs leads to the activation of the coagulation cascade. Inflammation caused by IFV infection increases various proinflammatory cytokines such as TNF-α, IL-1β, and IL-6 that induce the secretion of TF by ECs and monocytes (58). In addition to their roles in coagulation, activated proteins such as thrombin, FXa, and FVIIa also enhance the inflammatory response. The inflammatory potentiating abilities of coagulation factors are mediated through their activation of protease-activated receptors (PARs) that are expressed in platelets, ECs, macrophages, and respiratory epithelial cells (58). The TF/thrombin/PAR-1 pathway has been associated to the promotion of a deleterious innate inflammatory response to IFV infection in mice (64, 65).

Therefore, both the hyper-inflammatory response and the aberrant activation of coagulation, which are potentiated with each other, are involved in severe influenza pneumonia and are key events that have to be controlled in order to reach a favorable resolution of the infectious process.

## Beneficial Effects of Immunobiotics in IFV Immune-Coagulative Response

Considering the importance of the coagulative response in the outcome of influenza infection and the ability of immunobiotics to beneficially influence the immune response to this respiratory pathogen, we wonder whether some immunobiotic strains would be able to beneficially modulate the immuno-coagulative response triggered by IFV. For this purpose, we performed challenge-infection experiments in mice and evaluated the influence of viable and non-viable immunobiotic *L. rhamnosus* CRL1505 strain on the respiratory immuno-coagulative response induced by IFV (41, 42).

Our data demonstrated that oral administration of *L. rhamnosus* CRL1505 to mice significantly reduced lung viral titers and tissue damage after the challenge with IFV (41). We later explored the capacity of nasally administered *L. rhamnosus* CRL1505, alive or heat killed, to reduce the influenza burden of disease (42). Those treatments induced a significant decrease in IFV titers in lungs, lessened pulmonary damage, and increased survival. Interestingly, a similar effect was achieved with the nasal administration of viable and non-viable CRL1505 strain. Moreover, the nasal route was more efficient than the oral administration to protect mice against IFV infection (41, 42). The protective effect achieved by the immunobiotic strain was related to its ability to modulate the respiratory antiviral immune response, particularly to its capacity to improve the levels of IFN-γ and IFN-β in the respiratory tract (Figure 2). Type I IFNs trigger the activation of the JAK-STAT pathway and increase the expression of antiviral genes. In addition, IFN-γ is produced by immune cells, especially Th1 cells, and it further improves antiviral immune response by inducing activation of NK cells and macrophages. Therefore, the modulation of type I IFNs and IFN-γ would be responsible of the reduction of viral loads in IFV-infected mice previously treated with the CRL1505 strain, similarly to other immunobiotic strains as mentioned before (Table 1). We demonstrated that the CRL1505 strain increased the levels of gut CD3^+^CD4^+^IFN-γ^+^ T cells, induce a mobilization of these lymphocytes into the lung and enhanced the respiratory production of IFN-γ and the activity of local antigen presenting cells (41, 66, 67). It was also noted that nasal administration was more effective than the oral route to increase pulmonary CD3^+^CD4^+^IFN-γ^+^ T cells (41, 42). The mechanism by which nasally administered viable or heat-killed *L. rhamnosus* CRL1505 improves IFN-γ^+^ T cells population is not clear. However, our studies support the possibility that the immunobiotic strain *L. rhamnosus* CRL1505 impact in the nasal-associated lymphoid tissue or bronchial-associated lymphoid tissue producing an innate imprinting in antigen presenting cells that contribute to the enhanced number and activity of CD3^+^CD4^+^IFN-γ^+^ T cells.

Our studies also showed that immunobiotic treatments were able to beneficially modulate the activation of coagulation during respiratory viral infection, an effect that was not reported before (41, 42). Then, our studies were the first in demonstrating a beneficial modulation of the immune-coagulative response during respiratory TRL3 activation and IFV infection induced by immunobiotic microorganisms (Figure 3).

Although IFV is an ssRNA virus, it generates dsRNA replication intermediates that activate TLR3 and contribute to the initiation of the antiviral respiratory immune response. In fact, IFV triggers type I IFN secretion through TLR3 recognition in immune (myeloid DCs or macrophages) and non-immune (fibroblasts or pneumocytes) cells (68). Challenge-infection experiments with respiratory viruses in TLR3^−/−^ mice showed that TLR3 does not modify the clearance of viral pathogens but it is relevant for the modulation of the lung inflammatory response (69, 70). It was showed that wild-type mice mount a robust inflammatory response in the lung after IFV infection and that this process is significantly diminished in TLR3^−/−^ animals (70). TLR3^−/−^ mice showed a longer survival when compared wild-type animals and this effect was associated with a reduction of inflammatory cells recruitment and lower levels of inflammatory factors in the respiratory tract. Other *in vivo* studies also demonstrated that TLR3 activation by poly(I:C) enhanced proinflammatory cytokines and antiviral factors expression (71), altered vascular permeability (72), and incremented the levels of D-dimers indicating that coagulation and fibrinolysis were triggered. In line with these findings, it was observed that the levels of D-dimers in TLR3^−/−^ mice were significantly lower than in wild-type animals after poly(I:C) administration (63). In addition, by using siRNA technology it was demonstrated that TLR3 is a key receptor in the induction of the procoagulant state in ECs (63). Challenge of those cells with the TLR3 agonist poly(I:C) induced a decrease of TM and an enhancement of TF expression in a time- and dose-dependent manner. The results obtained in our own *in vivo* experiments were in line with these preceding reports (41, 42). We observed that three daily doses of nasally administered poly(I:C) to BALB/c mice induced a marked enhancement of inflammatory cells (neutrophils and macrophages) and proinflammatory mediators (IL-1β, TNF-α, IL-8, and IL-6) in the respiratory tract. Moreover, TLR3 activation also induced an increase in TF expression and thrombin–antithrombin complex (TATc) levels in the lung while it reduced TM expression. These inflammatory–coagulative modifications were accompanied by respiratory tissue alterations and impairment of lung function (41, 42).

Of interest, we demonstrated that orally (41) or nasally (42) administered immunobiotics before the challenge with poly(I:C) differentially modulated the inflammatory-coagulative response. *L. rhamnosus* CRL1505 was able to reduce and increase the expression of TF and TM, respectively, after the respiratory activation of TLR3. Thus, the CRL1505 strain significantly diminished coagulation activation in blood and in the respiratory tract after the nasal stimulation with poly(I:C).

We also evaluated pulmonary coagulation during IFV infection (41, 42). The respiratory virus induced activation of coagulation in the lungs of infected mice as demonstrated by the increased levels of respiratory TATc. These procoagulant changes were related to alterations in the expression of TM and TF in lungs. Our findings are in line with previous studies in humans and animal models of influenza infection demonstrating increased lung fibrin deposition and enhanced numbers of intravascular thrombi in the respiratory tract (59, 73, 74). We demonstrated that immunobiotic treatment is able to significantly diminish the activation of coagulation in IFV-challenged mice. In fact, lower levels of respiratory TATc and a reduced expression of TF was observed in *L. rhamnosus* CRL1505-treated mice infected with IFV when compared to controls (41, 42).

As mentioned before, IFV promote a procoagulant state directly through its capacity to infect ECs and monocytes stimulating the expression of TF (75, 76). In addition, IFV induce activation of coagulation indirectly by the enhancement of proinflammatory factors such as IL-6 (75, 76). Therefore, the ability of immunobiotics to modulate the IFV-triggered immune-coagulative response could be explained by their direct influence on viral replication related to the enhancement of the antiviral state in the respiratory mucosa, and indirectly through the modulation of the inflammatory response. Considering this last point, we performed experiments using anti-IL-10R blocking antibodies in order to evaluate the role of the regulation of the inflammatory response in the reduction of coagulation activation. Results showed that IL-10 is important for the regulation of coagulation induced by the immunobiotic *L. rhamnosus* CRL1505 (41). Blocking of IL-10R abolished the capacity of the CRL1505 strain to change the expression of TM and TF in the lungs. This was in line with our previous studies evaluating the ability of *L. rhamnosus* CRL1505 to confer protection against inflammatory damage induced by TLR3 activation or RSV infection, which showed that IL-10 is a key factor for the reduction of lung injury (67). Additionally, it was demonstrated that lethal disease caused by IFV infection is prevented by IL-10 administration through the reduction of lung immunopathology (77). Moreover, TF expression and procoagulant activity of macrophages and ECs are reduced by IL-10 (78, 79).

Therefore, we demonstrated that immunobiotic administration induce an early increase in the levels of TNF and IL-6 in the respiratory tract after poly(I:C), RSV, or IFV challenge, while the levels of those proinflammatory factors are significantly reduced later during infection (41, 42, 67). The early increase of proinflammatory mediators and the augmented levels of IFN-γ explain the ability of *L. rhamnosus* CRL1505 to diminish viral replication while the improved production of IL-10 would lead to a beneficial modulation of the immune-coagulative response which results in a reduced severity of lung damage. It has been suggested that respiratory viral infections increase the risk of venous thromboembolism and ischemic heart disease through ECs perturbation, coagulation activation, reduction of anticoagulant factors, and inhibition of fibrinolysis (80–82). Then, our studies suggest that immunobiotics could be an interesting alternative not only to reduce the incidence and/or severity of respiratory viral infections, but in addition to reduce the risk of atherothrombotic alterations associated to respiratory viral infections.

## Conclusion

Research from the last decade has clearly demonstrated that beneficial microorganisms are able to modulate respiratory tract immunity and promote the resolution and lessen the severity of respiratory infections caused by pathogens such as IFV. Studies in animal models have demonstrated that orally or nasally administered immunobiotics are able to improve protection against IFV by three main mechanisms. First, immunobiotics increase the respiratory antiviral state by their capacity to improve levels of type I IFNs, the number and activity of antigen presenting cells, NK cells, CD4^+^IFN-γ^+^ T, and IgA^+^ B lymphocytes, as well as the levels of systemic and mucosal specific antibodies. Second, immunobiotics beneficially modulate the IFV-triggered respiratory inflammatory response by inducing changes in the levels and kinetics of proinflammatory factors and immunoregulatory cytokines such as IL-10 that allow the clearance of virus with a minimal inflammatory lung tissue damage. Finally, as demonstrated by our recent research works, immunobiotics modulate lung immune-coagulative response triggered by TLR3 activation or IFV infection, mainly by down-regulating lung TF and restoring TM levels. Studies in animal models suggest that immunobiotics would influence principally the innate immune response, modulating in that way the early antiviral inflammatory response and the subsequent cellular and humoral immune responses. Therefore, immunobiotics would have mainly an adjuvant effect. However, the exact molecular mechanisms by which immunobiotics differentially modulate the innate antiviral immune response against IFV remain to be elucidated.

Additionally, a growing number of studies in humans have examined the effect of immunobiotics on the incidence and severity of IFV infection. Considering the impact of immunobiotics in the innate immune response clinical studies have evaluated principally their potential adjuvant effects on IFV vaccination (Table 2). Although mechanistic studies have not been addressed in depth, there is promising evidence for beneficial effects of immunobiotics on human respiratory health and resistance against IFV. These observations might be helpful to propose new preventive approaches to improve IFV control using immunobiotics by developing functional foods, pharmabiotics, or vaccine adjuvants.

**Table 2:** **Effect of probiotics on influenza virus (IFV) infection in humans**.

Strain	Viability	Route	Population studied	Effects	Reference
*Lactobacillus fermentum* CECT5716	Viable	Oral (capsule)	Randomized, double-blinded, and placebo-controlled human clinical trial in adults	Coadjuvant capability for anti-IFV vaccine. Lower incidence of influenza-like illness during 5 months after vaccination Increased proportion of NK cells, higher induction of Th1 cytokines and augmented specific T-helper and T-cytotoxic lymphocytes. Increased antigen specific IgA	(83)
*Lactobacillus casei* DN-114 001	Viable	Oral (fermented dairy drink Actimel^®^)	Randomized, multicentre, double-blind, and controlled studies in elderly population over 70 years of age	Coadjuvant capability for anti-influenza vaccine. Improved IFV-specific antibody titers after vaccination	(84)
*Lactobacillus* GG		Oral (capsule)	Randomized, double-blind, and placebo-controlled pilot study in adults	Coadjuvant capability for anti-IFV vaccine. Increased protective titer 28 days after vaccination for the H3N2 strain	(85)
*Lactobacillus plantarum* L-137	Non-viable (heat killed)	Oral (capsule)	Randomized, double-blind, and placebo-controlled pilot study in adults	Improved levels of interferon (IFN)-β before vaccination	(86)
*Bifidobacterium animalis* ssp. *lactis* BB-12w and *Lactobacillus paracasei* ssp. *paracasei* 431w	Viable	Oral (capsule and acidified dairy drink)	Randomized, double-blind, placebo-controlled, and parallel-group study in adults	Coadjuvant capability for anti-IFV vaccine. Improved vaccine-specific secretory IgA in saliva. Significant higher levels of vaccine-specific plasma IgG, IgG1, and IgG3	(87)
*Lactobacillus rhamnosus* strains GG and LC705	Viable	Macrophage stimulation	Human primary macrophages	Quantitative different IL-1β and type I IFN gene expression levels in macrophages. Diminished IFV replication and production of viral proteins in macrophages	(88)
*Lactobacillus pentosus* b240	Non-viable (heat killed)	Oral (tablet)	Randomized, double-blind, and placebo-controlled trial in elderly population over 65 years of age	Significant reduction of the incidence rate of the common cold	(89)
*L. paracasei* MoLac-1	Non-viable (heat killed)	Oral (jelly)	Randomized, double-blind, and placebo-controlled trial in elderly nursing home resident volunteers	Coadjuvant capability for anti-IFV vaccine. Improvement of hemagglutination inhibition titers against all different types of influenza antigens analyzed. Improvement in antibody titers against A/H3N2	(90)
*L. rhamnosus* GG	Viable	Oral (supplemented milk)	Randomized, double-blinded, and placebo-controlled in children of 2–6 years of age	Probiotic intervention did not reduce significantly the occurrence of the examined respiratory viruses, but the children that received the *GG* strain had fewer days with respiratory symptoms	(91)
*Lactobacillus brevis* KB290	Viable	Oral (fermented drink)	Open-label, parallel-group trial in children of 6–12 years of age	Reduced incidence of IFV infection in schoolchildren	(92)
*L. rhamnosus* GG	Viable	Oral (added to breast milk or formula)	Randomized, double-blind, and placebo-controlled trial in infants between the first and third days of life	Significant reduction in the incidence of viral respiratory tract infections	(93)
*Lactococcus lactis* ssp. *lactis* JCM5805	Viable	Oral (fermented dairy drink)	Randomized, placebo-controlled, and double-blind trial in adults	Significant decrease in major symptoms of influenza-like illness. IFN-α elicited by A/H1N1 on peripheral blood mononuclear cells prepared from volunteers tended to be higher, and IFN-stimulated gene 15 was significantly higher	(94)

## Author Contributions

HZ, SA, HK, and JV have designed, written, and revised the review article.

## Conflict of Interest Statement

The authors declare that the research was conducted in the absence of any commercial or financial relationships that could be construed as a potential conflict of interest.
